# An Adaptive Trajectory Clustering Method Based on Grid and Density in Mobile Pattern Analysis

**DOI:** 10.3390/s17092013

**Published:** 2017-09-02

**Authors:** Yingchi Mao, Haishi Zhong, Hai Qi, Ping Ping, Xiaofang Li

**Affiliations:** 1 College of Computer and Information, Hohai University, Nanjing 210098, China; zhonghs@hhu.edu.cn (H.Z.); qihai@hhu.edu.cn (H.Q.); amazingapple@hhu.edu.cn (P.P.); 2 School of Computer Information & Engineering, Changzhou Institute of Technology, Changzhou 213032, China; lixf@czu.cn

**Keywords:** mobile pattern analysis, spatio-temporal data, trajectory clustering, adaptive parameter calibration, grid

## Abstract

Clustering analysis is one of the most important issues in trajectory data mining. Trajectory clustering can be widely applied in the detection of hotspots, mobile pattern analysis, urban transportation control, and hurricane prediction, etc. To obtain good clustering performance, the existing trajectory clustering approaches need to input one or more parameters to calibrate the optimal values, which results in a heavy workload and computational complexity. To realize adaptive parameter calibration and reduce the workload of trajectory clustering, an adaptive trajectory clustering approach based on the grid and density (ATCGD) is proposed in this paper. The proposed ATCGD approach includes three parts: partition, mapping, and clustering. In the partition phase, ATCGD applies the average angular difference-based MDL (AD-MDL) partition method to ensure the partition accuracy on the premise that it decreases the number of the segments after the partition. During the mapping procedure, the partitioned segments are mapped into the corresponding cells, and the mapping relationship between the segments and the cells are stored. In the clustering phase, adopting the DBSCAN-based method, the segments in the cells are clustered on the basis of the calibrated values of parameters from the mapping procedure. The extensive experiments indicate that although the results of the adaptive parameter calibration are not optimal, in most cases, the difference between the adaptive calibration and the optimal is less than 5%, while the run time of clustering can reduce about 95%, compared with the TRACLUS algorithm.

## 1. Introduction

In recent years, with the rapid development of sensor technology and smart phones, GPS devices are widely applied to track moving objects, e.g., humans, vehicles, and animals, which can produce huge amounts of trajectory data every day. The trajectory data is the spatial-temporal data series from the moving objects with different timestamps. They contain a lot of information and help us understand the behaviors of the moving objects more directly. For example, zoologists can cluster the paths of animals to study the migration of animals [1]. Meteorologists explore the movement path of hurricanes through clustering and correlation analysis to improve the capabilities in disaster early warning and prevention [2]. Based on the clustering analysis of the movement patterns of vehicles, traffic managers can plan urban roads to mitigate the traffic jams [3,4]. For example, Yue et al. proposed the single-linkage clustering method to analyze taxi trajectory data to detect the time-dependent hot spots and movement patterns for urban traffic planning [5]. Moreover, a mobility-based clustering of vehicle trajectories was presented to detect hotspots and avoid the traffic jams [6].

Clustering analysis is one of the most important methods used in trajectory data mining. Trajectory clustering approaches can be applied in hotspot path analysis, mobility pattern analysis, and urban planning. At present, the trajectory clustering approaches include two types [7]: the first cluster the trajectory data based on the similarity of the full sequences. In other words, they take the whole trajectory as a unit to cluster the trajectory data. Those approaches have good effects on the clustering for the simple trajectories, however, they have negative effects for complex trajectories due to the fact they ignore the local detail sequences. The second type cluster the trajectory data based on the similarity of the sub-sequences. This means that the whole complex trajectory sequence is divided into several segments, which can be clustered with one segment as a unit. The second approaches have the ability to recognize the local features of complex trajectories.

Nonetheless, most available trajectory clustering algorithms depend on the calibration of one or multiple parameters. Meanwhile, the parameter values have a great influence on the effect of clustering. To reduce the complexity and workload of parameter calibration in trajectory clustering, a method called Adaptive Trajectory Clustering approach based on Grid and Density (ATCGD) is proposed in this paper. ATCGD firstly divides the trajectory data into multiple discrete segments through the average angular difference-based MDL (AD-MDL) algorithm. All of the discrete segments are mapped into the corresponding cells. Then, it calculates the average distance among the different segments in each cell, and the average number of the trajectory segments in each cell. Finally, adopting a DBSCAN-based approach, ATCGD carries out the adaptive parameter calibration based on the above data to realize effective and accurate trajectory clustering. As an illustration of the capabilities of the proposed method, we evaluate the performance of ATCGD approach on clustering quality and cost using two data sets from the random trajectories and hurricane trajectories in the Atlantic Ocean. The experimental results indicate that although the results of the adaptive parameter calibration are not optimal, in most cases, the difference between the adaptive calibration and the optimal one is less than 5%, while the run time of clustering can be reduced by about 95%.

The remainder of this paper is organized as follows: Section 2 discusses the related works and analyzes their drawbacks. The discrete trajectory partition algorithm, that is the average angular difference-based MDL (AD-MDL), is discussed in Section 3. Section 4 presents the proposed ATCGD approach, and the performance evaluations are given in Section 5. Discussion and conclusions are given in Section 6.

## 2. Related Work

### 2.1. Trajectory Clustering Approaches

Trajectory data can be regarded as time sequence data. Trajectory clustering is an important part of clustering analysis. To study the trajectory clustering of mobile objects, Gaffney et al. presented the mixture regression model-based trajectory clustering algorithm [8]. Furthermore, considering the temporal feature of trajectories, the spatial distance of the mobile objects was expanded to the spatial-temporal distance of the trajectories [9]. The time-focused trajectories clustering of moving objects algorithm, TFCTMO, was proposed based on the spatial-temporal distance. To obtain the moving cluster in the spatial-temporal trajectory data, the filter-based spatial-temporal clustering algorithm was discussed [10]. The filter-based cluster algorithm first filtered the trajectory data in the different time-scale ranges, and then clustered the data in the spatial-scale range within the same timestamp. All of the above clustering algorithms are based on the similarity of the full sequences.

Lee et al. thought that the clustering approaches based on the full sequences may have negative effects for complex trajectories due to the fact they ignore the local partial similarity [11]. Moreover, they put forward a partition-and-group framework and clustering algorithm—TRACLUS—that divides the whole trajectory into several segments and clusters them through the DBSCAN method [12,13,14]. The TRACLUS algorithm can recognize the local partial similarity of trajectories, however, in order to obtain good clustering quality, TRACLUS requires a large amount of workload to calibrate two parameters (the scanning range eps and the density minPts of each group). At the same time, the values of the two parameters are sensitive to the different data sets. In order to reduce the complexity and workload of parameter calibration, some parameter adaptive clustering algorithms based on the DBSCAN were put forward. For example, a self-adaptive density-based clustering algorithm (SA-DBSCAN) was presented in [15]. In the SA-DBSCAN approach, the distance of every object-pair in the data set is calculated as the input of two parameters eps and minPts. Although SA-DBSCAN can achieve good accuracy, it results in high computational complexity *O*(*n*^2^). Furthermore, through integrating the Affinity Propagation (AP) clustering method with DBSCAN, an AP-based clustering algorithm (APSCAN) was presented to cluster the objects without parameters [16]. However, the APSCAN algorithm still needs to compute the distance of every object-pair and thus exhibits high complexity. To further realize adaptive parameter calibration, the GCMDDBSCAN clustering algorithm established grid cells based on the various data, and then clustered the data based on optimal values of parameters eps and minPts with the cell as a unit [17].

From the above analysis, all of the DBSCAN-based clustering algorithms can achieve the adaptive parameter clustering for the simple object data. Considering the spatial and temporal characteristics of trajectory data, which differs from that of the simple object data, the trajectory clustering algorithm should reduce the computation complexity of clustering algorithms, especially in large-scale vehicle trajectories from intelligent systems. Based on the analysis of the DBSCAN-based clustering algorithms with adaptive parameter calibration, an Adaptive Trajectory Clustering approach based on Grid and Density (ATCGD) is proposed in this paper. ATCGD firstly divides the trajectory data into discrete trajectory segments based on the MDL-based method. All of the segments are mapped into the corresponding cells. Then, it calculates the average distance among the different segments in each grid cell, and the average number of the trajectory segments in each cell. Finally, adopting the idea of the DBSCAN-based method, ATCGD carries out the adaptive parameter calibration based on the above data to realize effective and accurate trajectory clustering.

Li et al. found that the existing trajectory algorithms focused on the static data and cannot deal with the problem of the data dynamic growth [18], so an incremental clustering framework of the trajectory, TCMM, was presented. In the TCMM framework, the whole trajectory was divided into several sequences and micro-clusters were established and dynamically maintained. The K-means method was also applied to the trajectory clustering problem [19]. However, it needed to determine the value of K in advance and cannot deal with noisy data, which results in poor performance in actual applications. Furthermore, the space covered by the trajectories was divided into cells. The trajectory clustering based on cells was proposed to cluster the grids when each cell is an object [20]. The cells-based clustering algorithm can exhibit good processing performance, while it ignores the differences among the sequences and leads to the poor clustering accuracy.

### 2.2. Trajectory Partition Methods

**Figure 1:**
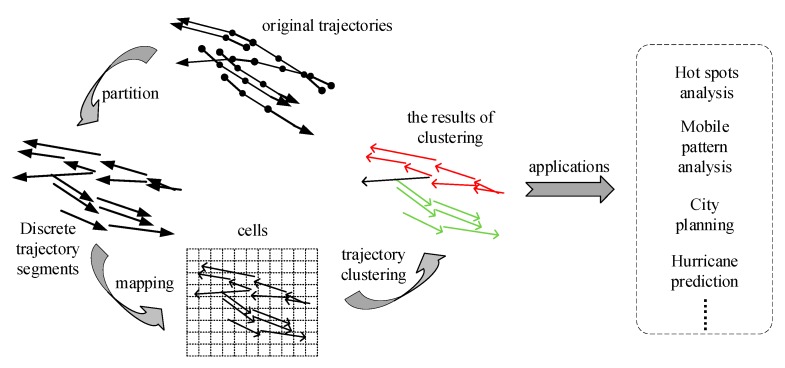
The illustration of the proposed ATCGD approach.

The proposed ATCGD algorithm includes three parts: partition, mapping, and clustering, as shown in Figure 1. In the partition phase, ATCGD applies the average angular difference-based MDL (AD-MDL) partition method to ensure the partition accuracy on the premise that it decreases the number of the segments after the partition. During the mapping procedure, the partitioned segments are mapped into the corresponding cells, and the mapping relationship between the segment and the cell are stored. In the clustering phase, adopting the DBSCAN-based method, the segments in the cells are clustered on the basis of the computed values of parameters from the mapping procedure. The clustering results can be applied in hotspot paths analysis, mobility pattern analysis, and urban planning.

In the field of trajectory partition, most of trajectory partition approaches rely on trajectory compression algorithms. The classical one is the Douglas-Peucker (DP) algorithm [21]. It detects some unnecessary points by calculating the information loss. Through introducing the concept of “window”, that is the segment, into the information loss computation, the OPening Window algorithm (OPW) was proposed [22]. OPW uses iterations to compress the trajectories with one “window” as one unit, instead of one whole trajectory as one unit. Using an iterations method, OPW can greatly reduce the computation cost. Afterwards, taking the time dimension into consideration, the Top-Down Time Ratio (TD-TR) algorithm was presented [12], and the optimal upper bound of errors compression algorithm (SQUISH-E) was proposed [23].

**Figure 2:**
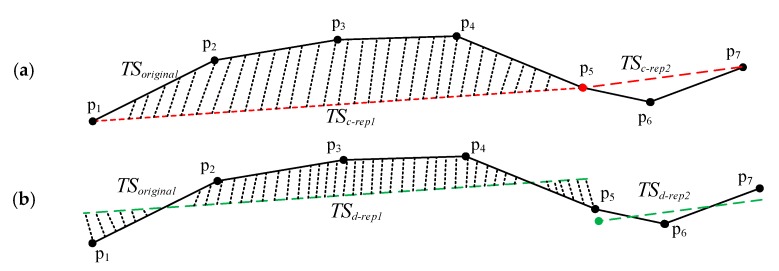
Illustration of the continuous representative segments and the discrete representative segments. (**a**) The continuous trajectory segments; and (**b**) The discrete trajectory segments.

These further improved the applicability of the compression algorithms in the GPS trajectory data. Lee et al. put forward the trajectory partition algorithm based on the Minimum Description Length (MDL) [11], which can effectively compress data as well as ensure the accuracy of the compressed data. From the above analysis of compression algorithms, it can be found that most of the compression algorithms try to obtain successive sequences of trajectory, which means all segments are end-to-end. However, continuity is unnecessary to the clustering of the trajectory segments. We can improve the accuracy of the compressed data when dealing with the discrete segments of trajectory. As shown in Figure 2a,b, $TS_{c-rep1}$ and $TS_{c-rep2}$ marked with the red line, are the continuous representative segments of the original trajectory data $TS_{original}$; and $TS_{d-rep1}$ and $TS_{d-rep2}$, marked with the green line, are the discrete representative segments, respectively. The dash area represents the area difference between the representative segments and the original trajectory. It is obvious that the area difference between $TS_{c-rep}$ and $TS_{original}$ is greater than that between $TS_{d-rep}$ and $TS_{original}$.

In this paper, adopting the AD-MDL discrete trajectories partition method, the proposed ATCGD trajectory clustering approach can map all of the segments into the corresponding cells. Then, based on the idea of the DBSCAN-based method, the segments are clustered through the calibration of adaptive parameters with the mapping relationship. The experimental results illustrate that the ATCGD approach can improve the effectiveness of clustering as well as ensure the accuracy.

## 3. Discrete Partitioning of Trajectories

### 3.1. Distance Measure Between the Trajectory Segments

**Definition 1** **(trajectory).** *With a given Euclidean space, a trajectory is composed of a series of trajectory points, expressed as* $TR=\text{\{}P_{1},\;P_{2},\;\ldots ,\;P_{n}\text{\}}$*, where the discrete trajectory points are sorted by timestamp, P_i_ refers to the trajectory point i,* $P_{i}=(x_{i},y_{i})$*, and n represents the number of points in the trajectory.*

**Definition** **2** **(sub-trajectory segment).** *Two adjacent discrete trajectory points* $P_{i}$ *and* $P_{i+1}$ *are connected to form a trajectory segment* $P_{i}P_{i+1}$*, which is a sub-trajectory segment, denoted as* $TS_{i}$*.*

A trajectory sequence consists of a series of discrete points. Two adjacent discrete points are connected to form a sub-trajectory segment. Due to the massive amount of trajectory data generated by mobile phones and other GPS equipment, the trajectory data compression is an important task for the sub-trajectory segments clustering. To reduce the workload of clustering all of the trajectory data, it should first partition the trajectory $TR=\text{\{}P_{1},\;P_{2},\;\ldots ,\;P_{n}\text{\}}$ into the multiple sub-trajectory segments $TS=\text{\{}TS_{1},\;TS_{2},\;\ldots ,\;TS_{n-1}\text{\}}$ by adopting the appropriate compression algorithm.

**Figure 3:**
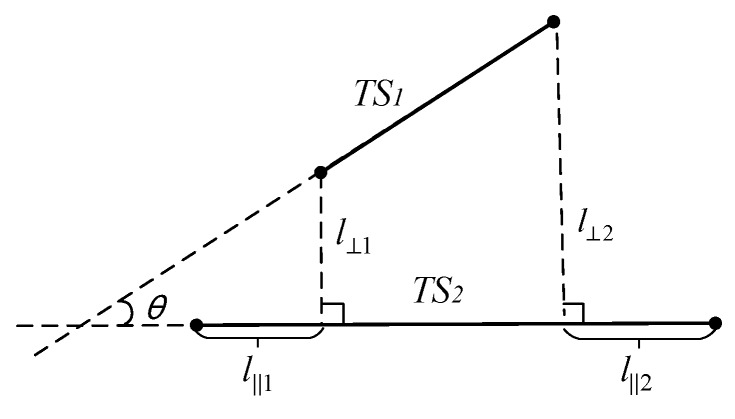
The illustration of distance measure between the two trajectory segments.

Lee et al. proposed a method to calculate the distance between two sub-trajectory segments with the weighted sum of the horizontal distance, vertical distance, and angular distance [10]. That distance of trajectory segments is suitable to the trajectory clustering. The horizontal distance can effectively avoid the noisy data problem when the distance between the two long trajectory segments is long. However, the angular distance may cause the problem of the short trajectory segments priority, which means that the shorter the trajectory segment is, the smaller the angular distance is. To solve the problem of the short trajectory segments priority, a new method to calculate the distance between the different segments is presented in this paper. As shown in Figure 3, $TS_{1}$ is the shorter trajectory segments and $TS_{2}$ is the longer one. $l_{⊥1}$ and $l_{⊥2}$ are the minimum and maximum vertical distance from any point in $TS_{1}$ to the segment $TS_{2}$, respectively. $l_{\|1}$ and $l_{\|2}$ are the distance from the corresponding intersection to the endpoint, respectively. $d_{⊥}$ is the vertical distance between the two segments calculated with $l_{⊥1}$ and $l_{⊥2}$. $d_{\|}$ is the horizontal distance between the two segments calculated with $l_{\|1}$ and $l_{\|2}$, θ is the angle between the two segments $TS_{1}$ and $TS_{2}$, as shown in Equations (1) and (2):

(1) d⊥={(l⊥1+l⊥22)×(1+sinθ)ifθ<π2(l⊥1+l⊥22)×(2+sin(θ−π/2))ifθ≥π2

(2) d‖={min(l‖1,l‖2)×(1+sinθ)ifθ<π2min(l‖1,l‖2)×(2+sinθ)ifθ≥π2

The distance between the two segments $TS_{1}$ and $TS_{2}$ can be computed as shown in Equation (3):

(3) $$dist(TS_{1},TS_{2})=dist(TS_{2},TS_{1})=d_{⊥}+d_{\|}$$

### 3.2. Discrete Representative Trajectory Segments

**Definition** **3** **(representative trajectory segments).** *Given a set of the trajectory segments* $TS=\text{\{}TS_{1},\;TS_{2},\;\ldots ,\;TS_{n}\text{\}}$*,* TS *can be represented with a trajectory segment* $TS_{rep}$ *as the representative trajectory segment.*

**Figure 4:**
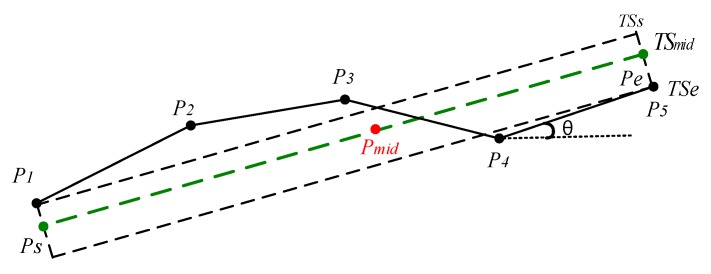
The diagram of the discrete representative trajectory segment.

According to the discussion in Section 2, from Figure 2a,b, it is obvious that the area difference between $TS_{c-rep}$ and $TS_{original}$ is greater than that between $TS_{d-rep}$ and $TS_{original}$. In order to reduce the area difference between the set of the partitioned segments and the original whole trajectory, ATCGD approach applies the discrete representative trajectory segments to replace the original whole trajectory, instead of the continuous representative trajectory segments. Figure 4 illustrates the discrete representative segments. As shown in Figure 4, $P_{i},\;i=1,\ldots ,5$ denotes the trajectory point in the original trajectory. $P_{mid}$ is the middle point in the original trajectory, where $x_{mid}=\sum_{i=1}^{5}x_{i}/5$, $y_{mid}=\sum_{i=1}^{5}y_{i}/5$.

In Figure 4, $TS_{mid}$ is the trajectory line through the middle point $P_{mid}$. Suppose that $TS_{i}\cdot \theta$ represents the clockwise angle between the trajectory segment $TS_{i}$ and the horizontal line, where $0\leq TS_{i}\cdot \theta <\pi$. $TS_{mid}\cdot \theta$ is the clockwise angle between the trajectory segment $TS_{mid}$ and the horizontal line. $TS_{mid}\cdot \theta$ can be calculated as follows:

(4) $$TS_{mid}\cdot \theta =\frac{\sum_{i=1}^{4}TS_{i}\cdot \theta }{4}$$

Then, it makes two vertical lines from two endpoints of original trajectory $P_{1}$ and $P_{5}$ to the line $TS_{mid}$, and intersects at the points $P_{s}$ and $P_{e}$, respectively. The trajectory segment $P_{s}P_{e}$ is just the representative trajectory segment of the original trajectory $\left\{P_{1},P_{2},P_{3},P_{4},P_{5}\right\}$, denoted as $TS_{rep}$. The coordinate values of the intersection $P_{s}$ can be calculated with Equation (5):

(5) $$\begin{array}{l} x_{s}=\frac{y_{1}+\tan(\pi /2-TS_{mid}\cdot \theta )\cdot x_{1}-y_{mid}+\tan(TS_{mid}\cdot \theta )\cdot x_{mid}}{\tan(TS_{mid}\cdot \theta )+\tan(\pi /2-TS_{mid}\cdot \theta )}\\ y_{s}=\tan(TS_{mid}\cdot \theta )\cdot (x_{s}-x_{mid})+y_{mid} \end{array}$$

In the same way, the coordinate values of the intersection $P_{e}$ can be calculated. It is obvious that the representative trajectory segments via the above method are discrete and cannot be end-to-end.

From Figure 2, the area difference between $TS_{c-rep}$ and $TS_{original}$ is greater than that between $TS_{d-rep}$ and $TS_{original}$. Therefore, this discreteness cannot take negative effect on the clustering results, instead it can generate the more accurate representative segments of the original trajectory.

To evaluate the accuracy of the representative trajectory segments, the cumulative distance difference between the discrete representative trajectory segment $TS_{rep}$ and the set of the original continuous segments $TS=\text{\{}TS_{1},\;TS_{2},\;\ldots ,\;TS_{n}\text{\}}$ is introduced, which is represented as φ. Because the vertical distance is one major impact factor on the difference between the representative trajectory segment and the original ones, the vertical distance is adopted to compute the cumulative distance difference, as shown in Equation (6):

(6) $$\varphi =\sum_{i=1}^{n}d_{⊥}(TS_{rep},TS_{i})$$

where n is the number of the original segments. The smaller φ is, the more accurate the representative trajectory segment is. Meanwhile, in order to verify the accuracy of the discrete representative trajectory segments, 1000 trajectories from the GeoLife data sets [24] are randomly selected. Assume that $\varphi_{discrete}$ represents the cumulative distance difference between the discrete representative segment and the original trajectory segment. $\varphi_{continuous}$ represent the cumulative distance difference between the continuous representative segment and the original one. The experimental results are that there are $\varphi_{discrete}\leq \varphi_{continuous}$ in the 982 trajectories from the 1000 trajectories, while there is only $\varphi_{discrete}>\varphi_{continuous}$ in the 18 trajectories. The experimental results indicate that the discrete representative trajectory segment can substitute the original one more accurately.

### 3.3. Discrete Trajectory Partition Algorithm

From daily life experience, we know that the trajectory variations of people’s or vehicle’s movements are always relatively smooth. That is to say, there are very small changes in the angle between the two adjacent trajectory segments. To further quantify the variations of trajectories, the average angular difference $Avg_{angle-diff}$ is introduced. Given a trajectory data $TR=\text{\{}P_{1},\;P_{2},\;\ldots ,\;P_{m}\text{\}}$, the average angular difference $Avg_{angle-diff}$ can be calculated as shown in Equation (7):

(7) $$Avg_{angle-diff}(1,n)=\sum_{i=2}^{n}\frac{\left|TS_{1}\cdot \theta -TS_{i}\cdot \theta \right|}{n-2},\mathrm{where}\;n>2$$

Lee et al. put forward the trajectory partition algorithm based on the Minimum Description Length (MDL) to compress data [8]. MDL is derived from Information Theory, which can be used to describe a given data set using fewer symbols than needed to describe the data literally. In essence, MDL can be applied to data compression. In the trajectory data compression, MDL can obtain a tradeoff between the number of sub-trajectory segments and the accuracy of the trajectories partition results, but MDL has high computational complexity to obtain the partitioned segments. In order to reduce the complexity of the trajectories partition, the average Angular Difference-based MDL (AD-MDL) is proposed to compress the trajectory data and partition the trajectories. AD-MDL consists of two phases: data filtering and trajectory partition.

In the data filtering phase, it eliminates the obvious outliers with the minimum cost based on the average angular difference $Avg_{angle-diff}$, which can reduce the computation workload during the trajectory partitioning. At first, the original trajectory data can be partitioned into multiple continuous segments. During the procedure of data filtering, the average angular difference $Avg_{angle-diff}$ is considered as the filtering factor. The filter threshold is $\theta_{threshold}$. For each continuous sub-trajectory segment, if its average angular difference is greater than the threshold $\theta_{threshold}$, the starting point of that sub-trajectory segment should be added into the set of the candidate trajectory points $TR_{c}$. Otherwise, the starting point of the segment is considered as an outlier and cannot be processed in the trajectory partition phase. After the data filtering, it can get the set of the candidate trajectory points $TR_{c}=\text{\{}Pc_{1},Pc_{2},\ldots ,Pc_{n}\text{\}}$. A GeoLife data set is introduced as an example to evaluate the performance on data compression. Based on the experimental results, it can be found that the AD-MDL can realize the 39% compression rate when the threshold value is $\theta_{threshold}=\pi /64$. Thus, it can greatly reduce the computation overhead in the trajectory partition phase.

In the trajectory partition phase, MDL method is still adopted to partition the compressed trajectories into discrete representative trajectories. During the data compression procedure, the overhead of MDL usually includes two parts: L(H) and L(D|H). H is the hypothesis, and D is the described data. L(H) is the overhead of describing the hypothesis and L(D|H) is the overhead to describe the D under the hypothesis H. MDL aims to find the optimal *H* to describe *D* to minimize the sum of L(H) and L(D|H). As to the trajectory partition, H is the set of discrete representative trajectory segments, and D is the original trajectory data. L(H) represents the total length of the all discrete representative segments. L(D|H) represents the difference between the discrete representative segments and the original trajectory. It is obvious that the greater number of the selected candidate points is, the more accuracy of the partition is. The greater L(H) is and the smaller L(D|H) is, which results in the high accuracy and high computation cost. Otherwise, it results in the low overhead and poor accuracy. When the sum of L(H) and L(D|H) is minimum, the trajectory partition can reach the tradeoff between the accuracy and computation cost. L(H) and L(D|H) can be computed as follows Equation (8):

(8) $$\begin{array}{l} L(H)=\sum_{i=1}^{m-1}\log(len(TS_{c_{i}-c_{i+1}}))\\ L(D|H)=\sum_{i=1}^{m-1}\sum_{j=c_{i}}^{c_{i}{}_{+1}-1}\log(d_{⊥}(TS_{c_{i}-c_{i+1}},P_{j}P_{j+1})) \end{array}$$

where $TS_{c_{i}-c_{i+1}}$ represents the discrete trajectory segment from the candidate point $Pc_{i}$ to $Pc_{i+1}$, $P_{j}P_{j+1}$ is the original trajectory segment in the $TS_{c_{i}-c_{i+1}}$, and $len(TS_{c_{i}-c_{i+1}})$ means the length of the discrete trajectory segment from the point $Pc_{i}$ to $Pc_{i+1}$.

To obtain the optimal trajectory partition, it should compute the global optimal solution to the minimum sum of L(H) and L(D|H), which results in the high computation overhead. To reduce the computation cost, we adopt a greedy solution to find the local optimal results to replace the global optimal results.

Suppose $Pc_{i}$ and $Pc_{j}$ are two candidate points from $TR_{c}=\text{\{}Pc_{1},Pc_{2},\ldots ,Pc_{n}\text{\}}$. $MDL(c_{i},c_{j})=L(H)+L(D|H)$ represents the minimum description length of part of trajectory segment $\text{\{}Pc_{i},Pc_{i+1},\ldots ,Pc_{j}\text{\}}$ and $c_{i}<c_{j}$. $L_{D}(c_{i},c_{j})$ represents the original trajectory length of the segment $\text{\{}Pc_{i},Pc_{i+1},\ldots ,Pc_{j}\text{\}}$, that is, $L_{D}(c_{i},c_{j})=\sum_{x=i}^{j-1}len(Pc_{x}Pc_{x+1})$. From the point $Pc_{i}$ as the starting point, if $MDL(c_{i},c_{j})<L_{D}(c_{i},c_{j})$, it reveals that all of the trajectory points in the segment $\text{\{}Pc_{i},Pc_{i+1},\ldots ,Pc_{j}\text{\}}$ are not trajectory characteristic points and the corresponding trajectory segment $\text{\{}Pc_{i},Pc_{i+1},\ldots ,Pc_{j}\text{\}}$ cannot be added into the set of the discrete representative segment, denoted as $D_{TS}$. Otherwise, the points in the segment $\text{\{}Pc_{i},Pc_{i+1},\ldots ,Pc_{j}\text{\}}$ are trajectory characteristic points and the corresponding segment can be transformed into the discrete representative segment with the Equations (4) and (5) discussed in Section 3.2.

According to the above discussion, the average Angular Difference-based MDL (AD-MDL) algorithm can be used to compress the trajectory data and create the discrete representative segments. The pseudo-code of the AD-MDL algorithm (Algorithm 1) is as given below. The AD-MDL trajectory partition algorithm contains two phases, the first one is the data filtering and the second one is to create the discrete representative trajectory segments. In the data filtering phase, part of the trajectory points is selected as the candidate point for the trajectory partition phase, based on the average angular difference. Thus, it can reduce the number of trajectory points to create the discrete representative segments and reduce the computation time in the second phase.

**Algorithm 1.** AD-MDL: The Average Angular Difference-Based MDL Trajectories Partition Algorithm.Input: Trajectory sequences $TR=\text{\{}P_{1},P_{2},\ldots ,P_{n}\text{\}}$, and the threshold of the average Angular Difference $\theta_{threshold}$Output: the set of discrete representative trajectory segments $D_{TS}$***// data filter phase***1: index = 1; $p_{start}=p_{1}$; $p_{start}$ is added into the set of candidate trajectory points $TR_{c}$2: for j=2 to n in the TR3: if $Avg_{angle-diff}(index,j)>\theta_{threshold}$ then4:   $p_{j}$ is added into the set $TR_{c}$5:   index=j; j=j+1;6: $p_{end}=p_{n}$;7: $p_{end}$ is added into the set $TR_{c}$***// trajectory partition phase***8: index =1; 9: for j=2 to m in the $TR_{c}$10:  if $MDL(c_{index},c_{j})>L_{D}(c_{index},c_{j})$11:    $TS_{c_{index}-c_{j}}$ is a discrete representative trajectory segment, and added into the set $D_{TS}$12:    index=j; j=j+1;13: end for14: return the set $D_{TS}$.


As shown in lines 1 to 7 of the AD-MDL algorithm, if the average angular difference is not greater than the threshold $\theta_{threshold}$, a new trajectory point is added. Otherwise, the new added trajectory point is the characteristic point and is added into the set of candidate points $TR_{c}$. In the trajectory partition phase, in order to obtain the clustering accuracy as well as the low complexity, the MDL-based method is adopted to create the discrete representative segments. As shown in the line 8 to line 14 of the AD-MDL algorithm, if there is $MDL(c_{index},c_{j})\leq L_{D}(c_{index},c_{j})$, the trajectory points between the $p_{c_{index}}$ and $p_{c_{j}}$ are non-characteristic points, and the successive point is included. If $MDL(c_{index},c_{j})>L_{D}(c_{index},c_{j})$, the trajectory points between the $p_{c_{index}}$ and $p_{c_{j}}$ are characteristic points and the corresponding segment $TS_{c_{index}-c_{j}}$ is added into the set of discrete representative segments $D_{TS}$. The AD-MDL algorithm traverses all of the trajectory points twice, so the computation complexity is O(n), where n is the total number of trajectory points.

## 4. Trajectory Clustering Based on Grid and Density

### 4.1. Grid Partition

We can get the discrete representative trajectory segments with the AD-MDL algorithm. After the trajectory partitioning, the partitioned segments should be mapped into the appropriate cells with the clustering method based on the grid and density, which is the task of the grid partition phase. The trajectory clustering based on the density should follow the principle of the cluster size from small to big. Suppose that the average number of the trajectory segments in each cell is represented as $Num_{avg}$. The value of $Num_{avg}$ should be as small as possible, which means that the average number of the trajectory segments should be minimum in each cell. However, in order to conduct the trajectories clustering based on the density, it needs to compute the distances among the different trajectory segments for each cell, which results in the heavy overhead of computation. In the experiments of Section 5.3, it can be found that the minimum value of $Num_{avg}$ cannot obtain the optimum of clustering. Through a lot of experiments, when $Num_{avg}=2$, it can obtain the best clustering quality.

**Figure 5:**
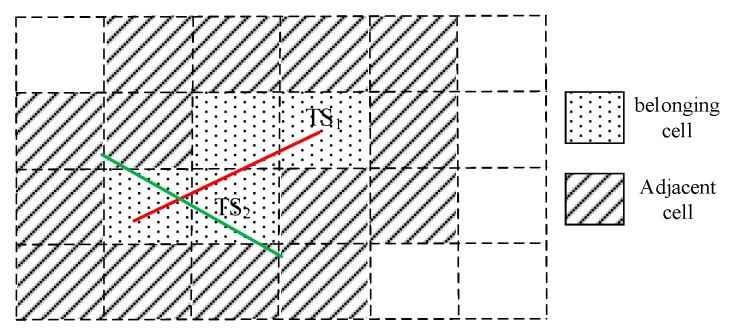
Schematic diagram of the belonged Cell and adjacent Cell.

**Definition** **4** **(belonging cell).** *The cells are passed by the trajectory segment* $TS_{i}$ *are defined as the belonging cells of* $TS_{i}$*, represented as* $Belong\_Cell.TS_{i}$*. The number of belonging cells of* $TS_{i}$ *is* $\left|Belong\_Cell.TS_{i}\right|$*. As shown in the* Figure 5*, the cells with point shaded are the belonging cells of the trajectory segment* $TS_{1}$*, and* $|Belong\_Cell.TS_{1}|=4$*.*

**Definition** **5** **(adjacent cell).** *The cells are adjacent to one of the belonging cells of the trajectory segment* $TS_{i}$ *are defined as the adjacent cells of* $TS_{i}$*, represented as* $Adjacent\_Cell.TS_{i}$*. The number of adjacent cells of* $TS_{i}$ *is* $\left|Adjacent\_Cell.TS_{i}\right|$*. As shown in the* Figure 5*, the cells with oblique lines shadow are the adjacent cells of the trajectory segment* $TS_{1}$*, and* $|Adjacent\_Cell.TS_{1}|=14$*.*

**Definition** **6** **(cell density).** *Suppose a certain cell* $cell_{i}$*, the number of the trajectory segments passing through the* $cell_{i}$ *is defined as the cell density of* $cell_{i}$*, denoted as* $cell_{i}.seg$*.*

During the procedure of the grid partition and mapping the trajectory segments into the corresponding cells, it needs to traverse every trajectory segment and recognize all of the belonging cells and adjacent cells of every segment, as well as every cell’s density. Those computation results are the inputs for the trajectory clustering.

### 4.2. Trajectory Clustering Algorithm

The DBSCAN-based clustering approaches should calibrate the values of two parameters eps and minPts. eps and minPts denote the radius of neighbor cells and the threshold of density of the trajectory segments, respectively. In Section 4.1, we could obtain the average distance among the different segments in each cell, and the average number of the trajectory segments in each cell. With the DBSCAN-based clustering approach, the ATCGD trajectory clustering approach carries out the adaptive parameters calibration eps and minPts, based on the above data to realize the effective and accurate trajectory clustering.

**Definition** **7** **(neighborhood of trajectory segment).** *Suppose there are two trajectory segments* $TS_{x}$ *and* $TS_{y}$ *in* $D_{TS}$*, that is* $TS_{x}\in D_{TS}$ *and* $TS_{y}\in D_{TS}$*, where* $D_{TS}$ *is the set of the discrete partitioned trajectory segments. If there has* $N_{eps}(TS_{x})=\{TS_{y}\in D_{TS}:dist(TS_{x},TS_{y})\leq eps\}$*, where* eps *is the radius of the neighbor cells,* $N_{eps}(TS_{x})$ *is the neighborhood of trajectory segment* $TS_{x}$ *with* eps*, denoted as* $N_{eps}(TS_{x})$*.*

From Definition 7, all of the trajectory segments, whose distance from the segment $TS_{x}$ is less than eps in the set $D_{TS}$, are the neighborhood of trajectory segment $TS_{x}$ with eps. The size of radius of the neighbor cells eps can determine the size of $N_{eps}(TS_{x})$ for the trajectory segment $TS_{x}$. Next, we will discuss the procedure of adaptive parameter calibration for eps.

It selects the cells with density greater than 1, that is $cell_{i}.seg>1$, where i=1,…,n, n is the number of cells. Suppose the number of the selected cells with $cell_{i}.seg>1$ is M, $cell_{i}.seg$ is the cell density of $cell_{i}$, $cell_{i}.TS_{x}$ is the trajectory segments $TS_{x}$ passing through $cell_{i}$. The radius of the neighbor cells eps can be computed as follows:

(9) EXPeps(i)=max(∑p=1celli.seg∑q=p+1celli.segdist(celli.TSp,celli.TSq))EXPavg=∑i=1MEXPeps(i)Meps=EXPavg+∑i=1M(EXPeps(i)−EXPavg)M

where $EXP_{eps}(i)$ is the expected value of eps for the $cell_{i}$, and $EXP_{avg}$ represents the average expected value of eps for all of the cells. From the discussion in Section 4.1, we set $Num_{avg}=2$ to obtain good clustering quality. Due to the value of $Num_{avg}$ is enough small, the distances among the different trajectory segments in each cell are very short. The maximum distance among the trajectory segments in the $cell_{i}$ is selected as the expected value of eps of the $cell_{i}$. The radius of the neighbor cells is the sum of the average expected value $EXP_{avg}$ and the standard deviation of all cells’ expected values. For any one cell $cell_{i}$, its cell density $cell_{i}.seg$ is constant. The computation complexity of eps is O(logn), where n is the number of the cells.

**Definition** **8** **(segment density).** *Suppose there is one trajectory segment* $TS_{x}$ *in* $D_{TS}$*, the density of* $TS_{x}$ *is defined as the number of trajectory segments in its neighborhood, denoted as* $\rho (TS_{x})$*. That is* $\rho (TS_{x})=|N_{eps}(TS_{x})|$*.*

**Definition** **9** **(core segment).** *Suppose there is one trajectory segment* $TS_{x}$ *in* $D_{TS}$*, and* minPts *is the threshold of density of the trajectory segments. If* $\rho (TS_{x})\geq minPts$*, the trajectory segment* $TS_{x}$ *is defined as the core segment of* $D_{TS}$*. Otherwise,* $TS_{x}$ *is non-core segment of* $D_{TS}$*. The set of core segments is denoted as* $D_{core}$ *and the set of non-core segments is denoted as* $D_{non-core}$*.*

In the ATCGD trajectory clustering approach, the threshold value of minPts is not fixed and may vary with the different number of the belonging cells of the trajectory segments. In the applications, if the density of the trajectory segment $TS_{x}$ is not less than the mean value through the statistical results, it can be considered that the density of the segment $TS_{x}$, $\rho (TS_{x})$, can meet the requirements of trajectory clustering. For the trajectory segment $TS_{x}$, the corresponding threshold minPts is set to $minPts=Num_{avg}\times |Belong\_Cell.TS_{i}|$. On the other hand, one trajectory segment may pass through one or more cells, and one cell can be covered by one or more trajectory segments. $Num_{avg}$ is the average number of the trajectory segments in each cell. $Num_{avg}$ can be further improved considering the many-to-many relationship between the $\left|Belong\_Cell.TS_{i}\right|$ and the $cell_{i}.seg$ for each segment and grid cell. The modified $Num_{avg}$ is denoted as $N_{avg}$ and can be computed as Equation (10):

(10) Navg=∑j=1n|Belong_Cell.TSj|n∑i=1Cnumcelli.segCnum

where $C_{num}$ is the number of the cells, and n is the total number of the trajectory segments.

**Definition** **10** **(directly density-reachable).** *Suppose there are two trajectory segments* $TS_{x}$ *and* $TS_{y}$ *in* $D_{TS}$*, that is* $TS_{x}\in D_{TS}$ *and* $TS_{y}\in D_{TS}$*. If* $TS_{x}\in D_{core}$ *and* $TS_{y}\in N_{eps}(TS_{x})$*,* $TS_{y}$ *are said to be directly density-reachable from* $TS_{x}$*. By Definition 10, no trajectory segments are directly density-reachable from a non-core segment.*

**Definition** **11** **(density-reachable).** *Suppose there are* m *trajectory segments in* $D_{TS}$*, that is* $TS_{1},TS_{2},\ldots ,TS_{m}\in D_{TS}$*, where* m≥2 *and* $TS_{1},TS_{2},\ldots ,TS_{m-1}\in D_{core}$*. If* $TS_{i}$ *is the directly density-reachable from* $TS_{i-1}$*, then* $TS_{m}$ *is the density-reachable from* $TS_{1}$*.*

The density-based trajectory clustering procedure includes three phases. The first phase is to map the discrete trajectory segments into the cell. Suppose there are n discrete representative trajectory segments obtained with the discrete trajectory partition algorithm (AD-MDL). $Num_{avg}$ is set to 2, and the area can be divided into $n/Num_{avg}$ cells. Then, it can calibrate two parameters eps and minPts to set the scanning radius of cells and the threshold of density of the trajectory segments and form a cluster, respectively, based on the Equations (9) and (10). The second phase is to execute the grid and density-based clustering with DBSCAN-based method. It starts with an arbitrary trajectory segment $TS_{i}$ that has not been visited. The $TS_{i}$’s neighborhood is retrieved, and if its density $\rho (TS_{i})$ is greater than minPts, a cluster is started. Otherwise, the trajectory segment is labeled as noise. If the trajectory segment $TS_{i}$ is found to be a dense part of a cluster, its neighborhood $N_{eps}(TS_{i})$ is also part of that cluster. All of the trajectory segments that are found within the neighborhood $N_{eps}(TS_{i})$ are added, as is their own neighborhood when they are also dense. This process continues until the density-reachable cluster is completely found. Then, a new unvisited trajectory segment $TS_{j}$ is retrieved and processed, leading to the discovery of a further cluster or noise. After the trajectory clustering, the set of the candidate clusters, $S_{cluster}$, are created. However, if one candidate cluster $C_{i}$ is not dense, which cannot meet the application’s requirement for the clustering quality. The last phase is to check the cardinality for each cluster. For one candidate cluster $C_{i}$, if the number of trajectory segments in the cluster $C_{i}$ is not greater than $\sum_{j=1}^{C_{num}}cell_{j}.seg/C_{num}$, where $C_{num}$ is the number of the cells, the cluster $C_{i}$ should be the final cluster and be removed from the set of the candidate clusters.

Based on the procedure of the density-based trajectory clustering, it can be found that a trajectory that is neither a core segment nor directly-reachable is called as a noise segment. A cluster should satisfy two properties: all trajectory segments within the cluster are mutually density-reachable; and if a trajectory segment is density-reachable from any segment of the cluster, it is part of the cluster as well.

The density-based trajectory clustering algorithm can be expressed in pseudo-code as follows.

**Algorithm 2.** The Density-Based Trajectory Clustering Algorithm.**Input:** The set of the discrete trajectory segments $D_{TS}=\{TS_{1},TS_{2},\ldots ,TS_{n}\}$**Output:** the clustering results**// map the trajectory segments into the cells**1: the area is divided into $n/Num_{avg}$ cells and the cells are covered by one or more than trajectory segments2: calibrate two parameters eps and $N_{avg}$ to set the radius and the threshold of segments density based on Equations (9) and (10)**// trajectory clustering based on the density**3: all of the trajectory segments in the $D_{TS}$ as unclassified and k=04: while ($D_{TS}\neq \emptyset$)5:   select any one trajectory segment $TS_{i}$ in the $D_{TS}$, and delete it6:   if ($TS_{i}$ is unclassified) then7:     $D_{tmp}=N_{eps}(TS_{i})$8:     if ($\left|N_{eps}(TS_{i})|\geq N_{avg}\times |Belong\_Cell.TS_{i}\right|$) then9:       $TS_{i}.cid=k$10:       while ($D_{tmp}\neq \emptyset$) // expand the current cluster11:         select any one trajectory segment $TS_{j}$ in the $D_{tmp}$ and delete it 12:         if ($\left|N_{eps}(TS_{j})|\geq N_{avg}\times |Belong\_Cell.TS_{j}\right|$) then13:           $D_{tmp}=D_{tmp}\cup N_{eps}(TS_{j})$14:         if ($TS_{j}$ is unclassified or noise) then15:           $TS_{j}.cid=k$16:    else17:      Mark $TS_{i}$ is noise18:   k=k+1**// check the trajectory cardinality**19: for each cluster $C_{i}$ in the set of clusters $S_{cluster}$20:   if ($|C_{i}|\leq \sum_{j=1}^{C_{num}}cell_{j}.seg/C_{num}$)21:     remove $C_{i}$ from the set $S_{cluster}$22: Return the final set of clusters.


From Algorithm 2, the density-based trajectory cluster algorithm includes three phases. From line 1 to line 2, the area is divided into the appropriate number of cells and the segments are mapped into the corresponding cells. Meanwhile, it executes the adaptive parameter calibration for eps and minPts. The complexity of the first phase is O(n). The clustering phase is from the line 3 to line 18, which adopts the DBSCAN-based method to cluster the discrete segments with the values of adaptive calibrated parameters, and get the candidate clusters. The complexity of clustering procedure is O(nlogn). To further check the results of clustering, it checks the density of each cluster. If the density of cluster is not greater than the average density, the candidate cluster should be removed, as shown from line 19 to line 22. As a whole, the complexity of the trajectory clustering based on the density is O(nlogn).

## 5. Performance Evaluation

### 5.1. Experimental Setup

**Figure 6:**
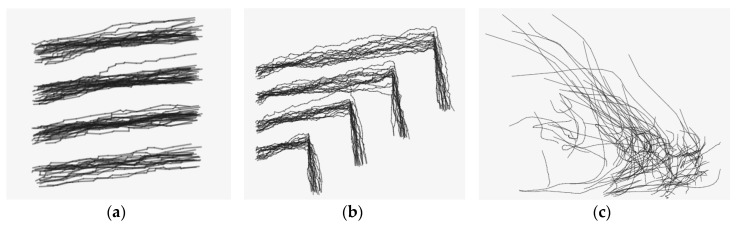
The trajectory in the RT and HT datasets. (**a**) RT1; (**b**) RT2; and (**c**) HT.

To evaluate the clustering performance of proposed trajectory cluster approach-ATCGD, two data sets are introduced. One is a series of randomly generated trajectories (hereafter referred to as Random Trajectory, RT), as shown in Figure 6a,b. The other is hurricane trajectory data in the Atlantic Ocean provided by American Weather Information System Company, referred to as Hurricane Track (HT) as shown in Figure 6c. RT data includes two patterns: RT1 and RT2. RT1 has about 100 trajectories and 2000 trajectory segments. Those trajectories can be clearly divided into four groups from top to bottom. RT2 has about 100 trajectories and 7000 trajectory segments, and is more complicated than RT1. The trajectories in RT2 are also divided into four groups. The trajectories in the RT1 and RT2 sets are similar to the trajectory data from vehicle movement, thus, RT1 and RT2 can represent a data set from a real application. The HT data set includes the hurricane track information about latitude, longitude, and the highest wind speed from 1851. The frequency of sampling is once for every 6 h. The experiments extract 100 hurricane trajectories with 2465 trajectory segments from 1940, which includes the latitude and longitude of the hurricane track.

To further evaluate the clustering quality of the proposed ATCGD approach, one metric QMeasure is introduced as the standard to evaluate the clustering effect [10]. QMeasure includes two parts: one is the sum of squared error (SSE) and the other is the penalty value of noise. The QMeasure can be calculated as follows:

(11) $$QMeasure=\sum_{i=1}^{Ncluster}(\frac{1}{2|C_{i}|}\sum_{x\in C_{i}}\sum_{y\in C_{i}}dist{\left(x,y\right)}^{2})+\frac{1}{2|D_{n}|}\sum_{p\in D_{n}}\sum_{q\in D_{n}}dist{\left(p,q\right)}^{2}$$

where $D_{n}$ is the noise set, $N_{cluster}$ is the number of the cluster of the trajectory segments, and $C_{i}$ represents the $i^{th}$ cluster of trajectory segments. $\left|C_{i}\right|$ is the number of the trajectory segments in the $i^{th}$ cluster. $\left|D_{n}\right|$ is the number of the noise trajectories. The sum of squared error (SSE) can be calculated with $\sum_{i=1}^{Ncluster}\left(\frac{1}{2|C_{i}|}\sum_{x\in C_{i}}\sum_{y\in C_{i}}dist{\left(x,y\right)}^{2}\right)$, which reflects the distances between the different trajectory segments in each cluster. The smaller value of eps is and the greater value of minPts is, it can obtain smaller SSE. In the applications, if it can calibrate appropriate values of two parameters eps and minPts, it can exhibit good cluster quality. At the same time, the noise trajectory data are considered when calculating the value of QMeasure. $\frac{1}{2|D_{n}|}\sum_{p\in D_{n}}\sum_{q\in D_{n}}dist{\left(p,q\right)}^{2}$ is used to calculate the sum of squared distances between the any noise trajectory segments, which is as the penalty. Therefore, the value of QMeasure and the quality of the clustering exhibits the negative correlation. The smaller the metric value of QMeasure is, the higher quality of the clustering is.

### 5.2. Clustering Performance

**Figure 7:**
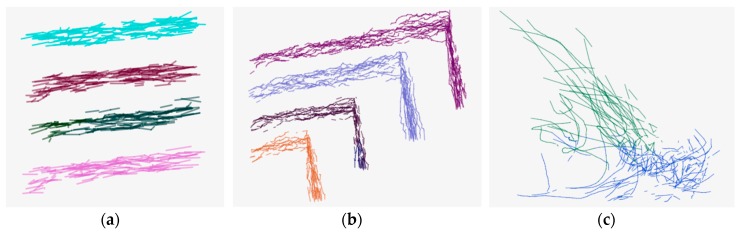
The clustering results on the RT and HT dataset, (**a**) RT1; (**b**) RT2; and (**c**) HT.

Figure 7 shows the clustering results with the RT1, RT2 and HT data sets, respectively. As shown in Figure 7, the different clusters are represented with different colors. From Figure 7a, the proposed ATCGD approach can cluster those trajectory data into four groups with high accuracy, which is in accordance with the expectation. Compared to the original trajectory data, it can be found that some trajectory segments are recognized as the noise. Figure 7b illustrates the clustering results with RT2. In contrast with RT1, the trajectories of RT2 exhibit apparent non-smoothness. This reveals that RT2 has greater difficulty than RT1 in clustering, but the ATCGD approach can still cluster those trajectories into four different groups. Therefore, the ATCGD can effectively be applied to the vehicle trajectory data, which has high similarity to the RT data set. Figure 7c shows the HT clustering results. From Figure 6c, the trajectories in the HT data set are much more complicated than those in the RT data set. The ATCGD approach can classify those hurricane data into two clusters, which conforms to the expectation. It implies that the ATCGD approach can also provide effective clustering for complex trajectory data.

### 5.3. Comparison Analysis

To further quantify the accuracy of the ATCGD clustering approach, we compare the ATCGD approach with TRACLUS in terms of QMeasure. Due to the slight differences about the distance calculation of the trajectory segments between the ATCGD and TRACLUS, it adopts the proposed distance computation equation between the trajectory segments in this paper, shown in Equation (3), to calculate QMeasure.

In the experiments, the different number of trajectories from RT and HT data sets are selected to evaluate the clustering quality. Thus 100, 200, 300, 400 hurricane track trajectories since 1940 from the HT data set are randomly selected and denoted as HT-100, HT-200, HT-300, and HT-400, respectively. Meanwhile, we apply the parameter calibration method proposed in the TRACLUS algorithm to conduct the experiments for twenty times and get the 20 different combination results of the two parameters eps and minPts. The minimum of combination results, that is the minimum QMeasure, is taken as the results of the TRACLUS algorithm. The experimental results of the ATCGD and TRACLUS algorithm are listed in Table 1.

From Table 1, it can be seen that the run time of TRACLUS algorithm is much higher than that of the ATCGD method. Meanwhile, the difference in the run times becomes greater between the two algorithms as the data size increases. The reason is that the ATCGD approach adopts the belonging cells and adjacent cells to determine the candidate set, which can be used to compute the neighborhood of eps. That method can greatly improve the efficiency and reduce the execution time of the trajectory clustering. The computation complexity of the ATCGD approach is O(nlogn) based on the analysis in the Section 4.2. On the contrary, without the index scheme, the computation complexity of the TRACLUS algorithm is up to $O(n^{2})$, where n is the number of trajectory points. On the other hand, as to the metric of the clustering quality QMeasure, the ATCGD approach does not appear to be much different from the TRACLUS algorithm. The ATCGD can obtain slightly better QMeasure than the TRACLUS algorithm. In most cases, the value of QMeasure in the ATCGD is smaller than that in the TRACLUS, except the HT-400 and RT2 data sets. The reason is that the ATCGD approach adopts the adaptive parameters calibration method to obtain the values close to the optimum, thus it can exhibit the good quality of clustering with the lower computation cost. While the TRACLUS algorithm can obtain the near-optimal combination results of two parameters eps and minPts through the large number of parameters calibrations, which results in the high accuracy and high computation complexity. If the combination results of two parameters are inappropriate, the TRACLUS algorithm will obtain the poor quality of trajectory clustering.

### 5.4. Parameter Sensitive Analysis

**Figure 8:**
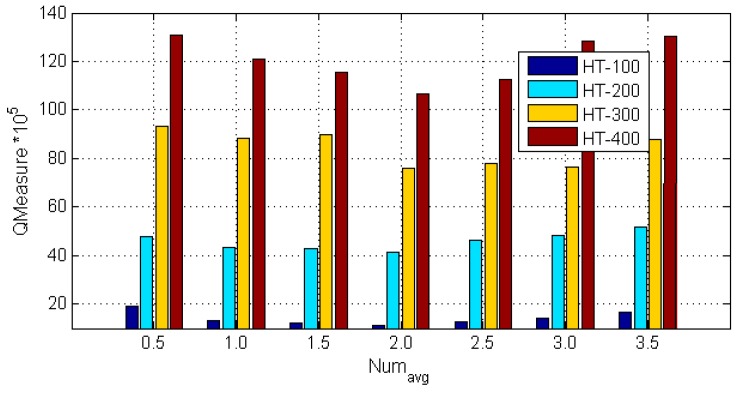
QMeasure values under different $Num_{avg}$.

In order to further provide the quantitative analysis of the parameter values of $Num_{avg}$, the HT-100, HT-200, HT-300, and HT-400 data sets are used to compute the quality of clustering metric QMeasure with the different values of $Num_{avg}$. The experimental results are shown in the Figure 8. When $Num_{avg}=2$, the value of QMeasure is minimum for all of the data sets. When $Num_{avg}<2$, the value of QMeasure decreases with the increase of $Num_{avg}$. On the contrary, when $Num_{avg}>2$, the value of QMeasure increases with the increase of $Num_{avg}$. Based on the experimental results, when the ATCGD approach sets $Num_{avg}$ to 2, it can get better quality of the trajectory clustering.

**Figure 9:**
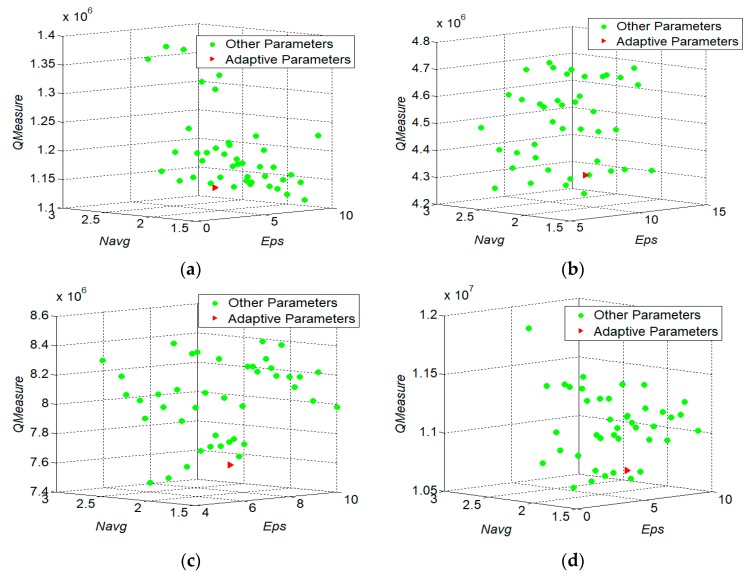
Experimental results of parameter adaptive analysis, (**a**) HT-100; (**b**) HT-200; (**c**) HT-300; and (**d**) HT-400.

To verify the correctness of the parameters calibration, two parameters eps and $N_{avg}$ (minPts can be computed based on $N_{avg}$) are selected for the sensitivity analysis. The data sets are still HT-100, HT-200, HT-300, and HT-400. We compare the different values of QMeasure with different combination of eps and $N_{avg}$ as well as the adaptive calibration values of those two parameters $eps_{a}$ and $N_{avg\_a}$. The value range of is $\left[\left\lfloor eps_{a}-3\right\rfloor ,\left\lfloor eps_{a}+3\right\rfloor \right]$ and the step is 1. The value range of $N_{avg}$ is $\left[N_{avg\_a}-0.6,N_{avg\_a}+0.6\right]$ and the step is 0.2. Figure 9 illustrates the distributions of QMeasure with different values of eps and $N_{avg}$ in the data sets of HT-100, HT-200, HT-300, and HT-400. As shown in Figure 9, the red points are the results of adaptive parameter calibration for $eps_{a}$ and $N_{avg\_a}$; the green points are the results of different combinations with different values of eps and $N_{avg}$

From Figure 9, it can be found that there is a large variation range of QMeasure with the different combinations of two parameters’ values, when adopting the TRACLUS algorithm. While the ATCGD approach can get the small value of QMeasure. The reason is that it adopts the adaptive parameters calibration method to compute the value of QMeasure. On the other hand, if the difference between the values of QMeasure by adopting the adaptive parameters calibration and the optimal combination is smaller, the ATCGD approach can obtain higher quality of trajectories clustering. Moreover, although the results of the adaptive parameter calibration are not optimal, in most cases, the difference between the values of QMeasure with the adaptive calibration and the optimal combination is less than 5%. It indicates that the adaptive calibrated parameters eps and $N_{avg}$ can gain good clustering effects.

## 6. Conclusions

Clustering analysis is one of the most important issues in trajectory data mining. Trajectory clustering can be widely applied in hotspots detection, mobile pattern analysis, urban transportation control, hurricane prediction, etc. Many trajectory clustering algorithms have been proposed to obtain good clustering performance. Nonetheless, most available trajectory clustering algorithms depend on calibration of one or multiple parameters. Meanwhile, the values of these parameters have a great influence on the effect of clustering. To reduce the complexity and overhead of parameter calibration in trajectory clustering, an Adaptive Trajectory Clustering approach based on Grid and Density, ATCGD, was proposed in this paper. ATCGD firstly divides the trajectory data into multiple discrete segments through the proposed the average angular difference-based MDL (AD-MDL) algorithm. All of the discrete segments are mapped into the corresponding cells. Then, it calculates the average distance among the different segments in each cell, and the average number of the trajectory segments in each cell. Finally, adopting a DBSCAN-based approach, ATCGD carries out an adaptive parameter calibration based on the above data to realize effective and accurate trajectory clustering. With two data sets from random trajectories and hurricane trajectories on the Atlantic Ocean, we evaluate the performance of the ATCGD approach on clustering quality and cost. The experimental results indicate that although the results of the adaptive parameter calibration are not optimal, in most cases, the difference between the adaptive calibration and the optimal is less than 5%, while the run time of clustering can be reduced by about 95%.

## Figures and Tables

**Table 1:** Comparison of clustering quality between ATCGD and TRACLUS.

	TRACLUS		ATCGD	
	*QMeasure*	Run Time (s)	*QMeasure*	Run Time (s)
HT-100	1,486,875	1.25	1,140,856	0.14
HT-200	5,416,222	5.84	4,327,626	0.23
HT-300	8,164,510	15.75	7,602,455	0.44
HT-400	9,741,195	26.34	10,682,513	0.61
RT1	461,437	1.07	39,426	0.09
RT2	164,351	21.75	176,269	0.57
